# Speech Enhancement of Mobile Devices Based on the Integration of a Dual Microphone Array and a Background Noise Elimination Algorithm

**DOI:** 10.3390/s18051467

**Published:** 2018-05-08

**Authors:** Yung-Yue Chen

**Affiliations:** Department of Systems and Naval Mechatronic Engineering, National Cheng Kung University, Tainan City 701, Taiwan; yungyuchen@mail.ncku.edu.tw; Tel.: +886-275-7575 (ext. 63541)

**Keywords:** speech enhancement, estimator design, background noise reduction, mobile device

## Abstract

Mobile devices are often used in our daily lives for the purposes of speech and communication. The speech quality of mobile devices is always degraded due to the environmental noises surrounding mobile device users. Regretfully, an effective background noise reduction solution cannot easily be developed for this speech enhancement problem. Due to these depicted reasons, a methodology is systematically proposed to eliminate the effects of background noises for the speech communication of mobile devices. This methodology integrates a dual microphone array with a background noise elimination algorithm. The proposed background noise elimination algorithm includes a whitening process, a speech modelling method and an *H*_2_ estimator. Due to the adoption of the dual microphone array, a low-cost design can be obtained for the speech enhancement of mobile devices. Practical tests have proven that this proposed method is immune to random background noises, and noiseless speech can be obtained after executing this denoise process.

## 1. Introduction

Currently, the speech communication function of mobile devices has been well-designed and widely used as a convenient tool for contacting others due to its portable characteristics. However, the speech communications of mobile devices are inevitably affected by varying environment noises. Several published speech enhancement algorithms based on a microphone have been studied in the past two decades for tackling the elimination of background noises due to their realization convenience [1,2,3,4]. From the revealed facts, single microphone designs obviously could not provide effective speech enhancement in highly non-stationary noise environments to decouple the desired speech and random noises via using one measurement of raw data.

Background noise reduction designs utilizing array-based microphone configurations have become the main trend due to the aforementioned reason. By increasing the number of microphones, array-based designs outperform satisfactory speech enhancement performances with respect to designs with only one microphone. While array-based designs provide promising effects in reducing background noises, they encounter serious challenges in practical mobile device applications, which always take total cost, size, weight, and so on into account. Dual microphone array designs perhaps are the remedy which can meet these requirements.

From mathematical expressions, existing background noise reduction algorithms using dual-microphone array designs can be separated into two categories: (1) spectrum domain expression and (2) time domain expression. The most popular method for speech enhancement designs of microphone arrays in spectrum domain is beamformer designs [5,6,7]. The speech enhancement achievements of these adaptive filters are acceptable, but these kinds of speech enhancement designs have two major disadvantages: (1) locations of the main speech and background noises, including reflections and reverberations must be fixed for achieving better background noise reduction performance in real environments, and (2) the spectrum of the passed noises must be predicted in advance. Another spectrum domain method often utilized to treat the speech enhancement problem is the power level difference method (PLD). Depending on the precise estimation of the power spectral densities of speeches, better speech enhancement performances can be obtained using this method. For solving drawbacks of conventional power level difference methods, a modified version which can precisely estimate the stochastic property of speech with optimal thresholds is investigated [8]. However, time-varying optimal thresholds of this method are not easily calculated in practice because the real-time characteristic of the microphone array which cannot be measured online has a strong influence on the PLD.

The Kalman filter represented in state-space formulation has been utilized for speech enhancement problems in the past year [9]. In Kalman filter design, the background noise is strictly assumed as a signal with a zero mean and a known covariance. Due to this assumption, the Kalman filter inherently possesses no noise reduction capability with regard to the colored background noise reduction problem. For improving the drawback of the Kalman filter algorithm with respect to the colored background noises, a modified version was proposed [10]. The main characteristic of this modified version is the searching of non-speech frames, and the purpose is to calculate the noise covariance. Theoretically, detecting no-speech frames offline is possible. However, it is very difficult to promptly identify the statistical occurrence of noises in the real environment. The above depictions point out the guideline to effectively enhance speech quality when mobile devices are used in the presence of colored noises: “An estimator without knowing the statistics of the background noises is necessary”. Several robust estimators are investigated for background noise elimination designs in the past decade [11,12,13,14]. By following the guideline, the steady state form of the Kalman filter known as the *H*_2_ estimator combined with a colored noise whitening process is studied for the speech enhancement problem in this investigation. By integrating this modified *H*_2_ estimator with a dual microphone array, an effective background noise reduction design that has low calculation power consumption and can effectively mitigate the non-stationary background noises is developed. The overall speech enhancement process of this investigation can be summarized as the following: Step 1, two measured data pairs of microphone channels are whitened as a white noise driven speech sequence. This speech sequence is mathematically expressed as a corresponding difference equation which contains a set of identifiable parameters in Step 2 and further formulated as a vector-matrix form in time domain for conveniently deriving the related *H*_2_ estimator in Step 3. From real tests, this proposed method can effectively remove the unknown background noises and deliver promising speech enhancement performances in severe scenarios.

## 2. Speech Enhancement Process for Mobile Devices

### 2.1. Introduction of Overall Speech Enhancement Process

**Figure 1:**
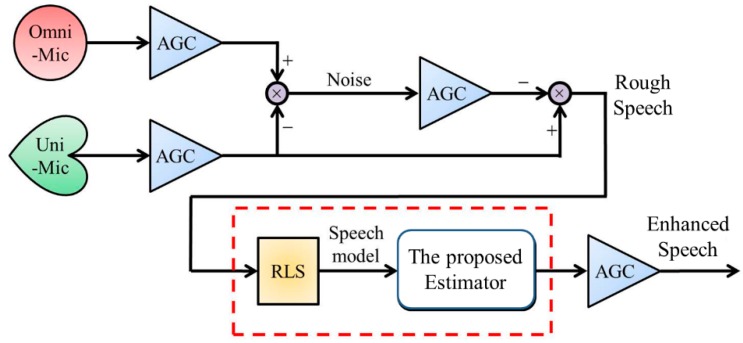
The schematic of the proposed speech enhancement process.

The overall speech enhancement process of mobile devices is depicted as Figure 1. There are four parts in this process: (1) a dual-microphone array, (2) an auto gain control process (AGC) [15], (3) a system identification algorithm for the real-time speech modeling, and (4) an optimal *H*_2_ estimator. The dual microphone array constructed with an omnidirectional microphone (Omni-Mic) and unidirectional microphone (Uni-Mic) is used for collecting all possible sound sources. AGC process after the dual microphone array is utilized to compress or amplify the intensities of the measured sound signals equally. The whitened speech model is mathematically identified by the Recursive Least-Squares algorithm (RLS) [16] and represented as a vector-matrix form. Based on the vector-matrix form, an *H*_2_ estimator is proposed to mitigate the effects of the residual of background noises and deliver enhanced speech. In the following, details of each part will be addressed.

**Remark** **1.** *As an inherent characteristic, omnidirectional microphones can pick up sounds equally from all directions of the surrounding environment. However, unidirectional microphones only pick up sounds with high gain from a specific side in order to get good gain on the recording.*

**Figure 2:**
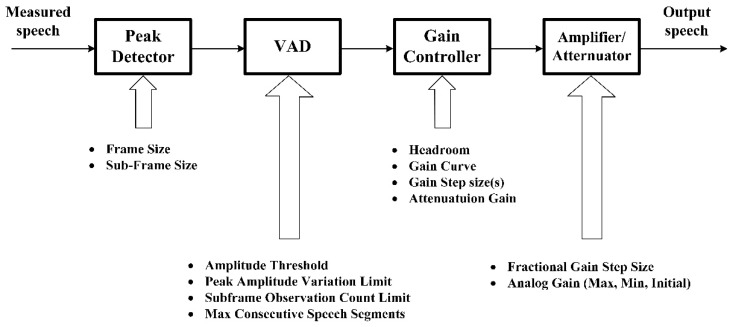
Detailed process of the AGC.

**Remark** **2.** *Generally, the AGC contains a peak detector, voice activity detector (VAD), gain controller, and amplifier/attenuator as in Figure 2. Function of the peak detector is to detect the peak signal envelope. As to the VAD, it is used to detect the character of a sub-frame of the input signal (speech or noise). The gain controller plays the role to provide the required gain to enhance the speech signal amplitude. By changing the analog gain of the analog to digital converter, the Amplifier/Attenuator can adjust the speech signal amplitude.*

### 2.2. Background Noise Whitening and Extraction of a Rough Speech

As Figure 1 shows, a rough speech before RLS can be obtained via using the measured raw data of the dual microphone array. This is a semi-hardware whitening process, and this pretreated speech reserves minor background noises but is cleaner than outputs of the dual microphone array. As mentioned above, the whitening process of colored background noises in Figure 1 can be described as below in detail.

The measured raw data of the dual microphone array (Omni-Mic and Uni-Mic) can be expressed as follows:

(1) $$\Xi =\left\{\left[x_{omni}(t),x_{uni}(t)\right]\in {ℜ}^{2}|1\leq t\leq K\right\}$$

where Ξ is the data set of measured raw data of Omni-Mic (*x_omni_*(*t*)) and Uni-Mic (*x_uni_*(*t*)) at time *t*. *K* is the length of total sampling raw data.

The difference of the measured raw data of Omni-Mic (*x_omni_*(*t*)) and Uni-Mic (*x_uni_*(*t*)) can be obtained by using the following subtraction:

(2) $$x_{e}(t)=x_{omni}(t)-x_{uni}(t),\;\mathrm{for}\;1\leq t\leq K$$

Collecting the difference *x_e_*(*t*) in Equation (2), a background noise data set $\Xi_{e}=\left\{x_{e}(t)\in ℜ|1\leq t\leq K\right\}$ can be obtained via this subtraction. From Equation (2), a large number of the background noises are obviously reserved in the data set Ξ*_e_*, and this set can be regarded as a set of background noises. By using the similar subtraction manipulation for the measured raw data of Uni-Mic (*x_uni_*(*t*)) and *x_e_*(*t*) in the set of background noises Ξ*_e_*, a rough speech with a small number of background noises yields at time *t* as follows:

(3) $$r_{s}(t)=x_{uni}(t)-x_{e}(t)$$

*r_s_*(*t*) in Equation (3) reserves a large number of speech and less background noises. Collecting raw data *r_s_*(*t*), a set for the whitened speech can be defined as $\Xi_{S}=\left\{r_{s}(t)\in ℜ|1\leq t\leq K\right\}$. Based on this whitened speech data set $\Xi_{S}$, an autoregressive (AR) model can be used to represent the character of the collected data *r_s_*(*t*).

The AR model of the slight noised speech in Equation (3) can be expressed as below:

(4) $$r_{s}(t)=\sum_{i=1}^{n}a_{i}r_{s}(t-i)+w_{s}(t)$$

(5) $$y(t)=r_{s}(t)+v_{s}(t)$$

where *r_s_*(*t*) is the rough speech in Figure 1 obtained from the whitening process in Equations (1)–(3), and *n* is the system order. *w_s_*(*t*) and *v_s_*(*t*) are modeling uncertainty and sensor noise and are uncorrelated with *r_s_*(*t*) and with zero means. *a_i_*, for *i* = 1, …, *n* are identifiable parameters. *y*(*t*) is the noisy measurement output and is the rough speech *r_s_*(*t*) in Figure 1 corrupted with a sensor noise *v_s_*(*t*).

For finding the identifiable parameters *a_i_*, for *i* = 1, …, *n*, Equation (4) can be expressed as the following regression form:

(6) $$r_{s}(t)=\lambda {\left(t\right)}^{T}\hat{\Theta }(t)+w_{s}(t)$$

where $\lambda (t)={\left[\begin{array}{ccc} r_{s}(t-1) & \cdots & r_{s}(t-n) \end{array}\right]}^{T}$ is the real-time raw data vector, and $\hat{\Theta }(t)={\left[\begin{array}{ccc} a_{1} & \cdots & a_{n} \end{array}\right]}^{T}$ is the identifiable parameter vector. The parameter vector $\hat{\Theta }(t)$ is capable of being identified by several system identification methods. In this investigation, the Recursive Least-Squares identification algorithm (RLS) algorithm [16] will be adopted later to iteratively search for the optimal values for the parameter vector $\hat{\Theta }(t)$.

### 2.3. System Identification: Recursive Least-Squares Identification Algorithm

In practice, the input and output signals can be obtained in every experiment, but the parameter vector $\hat{\Theta }(t)={\left[\begin{array}{ccc} a_{1} & \cdots & a_{n} \end{array}\right]}^{T}$ of the difference equation in Equation (4) are unknown; hence the parameters of difference equation should be identified optimally by using the input and output signals. As depicted in the above, RLS will be utilized to calculate the real-time parameters of the difference equation in Equation (4). By fitting the pair of measured input and output speech data, a selected difference equation with optimal parameters can be obtained. RLS algorithm is expressed as the following:

(7) $$e(t)=y(t)-\lambda {\left(t\right)}^{T}\hat{\Theta }(t-1)$$

(8) $$P(t)=\frac{1}{f}\left(P(t-1)-\frac{P(t-1)\lambda (t)\lambda {\left(t\right)}^{T}P(t-1)}{f+\lambda {\left(t\right)}^{T}P(t-1)\lambda (t)}\right)$$

(9) $$\hat{\Theta }(t)=\hat{\Theta }(t-1)+P(t)\lambda (t)e(t)$$

where *P*(*t*) is a time varying coefficient covariance at the time instant of *t*, $\hat{\Theta }(t)$ is parameter vector, *λ*(*t*) is input speech data vector, *e*(*t*) is the real-time output error vector, *y*(*t*) is the measurement output. *f* is defined as the forgetting factor and can be selected from the range [0.95,1] for the real implementation.

By using RLS algorithm in Equations (7)–(9), $a_{i}$ of $\hat{\Theta }(t)$ are identified optimally. The slight noised speech model in Equations (4)–(5) can be formulated as the following vector-matrix form:

(10) $$R(t+1)=A_{s}R(t)+B_{s}w_{s}(t)$$

(11) $$y(t)=C_{s}R(t)+D_{s}v_{s}(t)$$

$$A_{s}=\left[\begin{array}{ccccc} a_{1} & a_{2} & \cdots & a_{n-1} & a_{n}\\ 1 & 0 & \cdots & \cdots & 0\\ 0 & \ddots & \ddots & \ddots & \vdots \\ \vdots & \ddots & \ddots & \ddots & \vdots \\ 0 & \cdots & 0 & 1 & 0 \end{array}\right]\in {ℜ}^{n\times n},B_{s}=\left[\begin{array}{c} 1\\ 0\\ 0\\ \vdots \\ 0 \end{array}\right]\in {ℜ}^{n\times 1},C_{s}={\left[\begin{array}{c} 1\\ 0\\ 0\\ \vdots \\ 0 \end{array}\right]}^{T}\in {ℜ}^{1\times n},D_{s}=1$$

where $R(t)={\left[\begin{array}{ccccc} r_{s}(t) & r_{s}(t-1) & \cdots & r_{s}(t-n+2) & r_{s}(t-n+1) \end{array}\right]}^{T}$ is the state vector, and $w_{s}(t)$ and $v_{s}(t)$ are instances of white noise.

Based on the white noise driven speech model in Equations (10) and (11), an optimal estimator will be derived in the following.

### 2.4. H_2_ Estimator Design

In this section, the steady state form of the Kalman filter named: *H*_2_ Estimator will be derived, and this is an optimal estimator. Assume ϒ(*t*) is the desired speech to be reconstructed from Equations (10) and (11):

(12) ϒ(t)=ΔR(t)

where Δ is a constant matrix, which is arranged to extract the desired signal ϒ(*t*) from the state vector *R*(*t*). The object of developing the *H*_2_ estimator is to optimally estimate $\hat{ϒ}(t)$ via using the measured noisy output signal *y*(*t*). For reconstructing the original clean speech, the *H*_2_ estimator can be formulated in state space form as:

(13) $$\begin{array}{l} \hat{R}(t+1)=A_{s}\hat{R}(t)+L_{2}\left[y(t)-C_{s}\hat{R}(t)\right]\\ \;\hat{ϒ}(t)=\Delta \hat{R}(t) \end{array}$$

where $L_{2}\in {ℜ}^{n\times 1}$ is the *H*_2_ estimation gain of the steady state *H*_2_ estimator in Equation (12) and will be derived below.

The estimation error of the desired speech ϒ(*t*) and the estimated $\hat{ϒ}(t)$ can be calculated by subtracting ϒ(*t*) and $\hat{ϒ}(t)$ as:

(14) $$\begin{array}{l} {ϒ}_{e}(t)=ϒ(t)-\hat{ϒ}(t)\\ \;=\Delta \tilde{R}(t) \end{array}$$

where $\tilde{R}(t)=R(t)-\hat{R}(t)$ is the state estimation error.

### 2.5. Estimation Gain L_2_ of H_2_ Estimator

The *H*_2_ performance index for the speech enhancement problem of mobile devices with a dual microphone array can be expressed by the mean-square error of estimation error ${ϒ}_{e}(t)$ [17] as:

(15) $$\begin{array}{ll} J & =E\left\{{ϒ}_{e}(t+1){ϒ}_{e}{\left(t+1\right)}^{T}\right\}\\ & =E\left\{\Delta \tilde{R}(t+1)\tilde{R}{\left(t+1\right)}^{T}\Delta^{T}\right\} \end{array}$$

where the estimation error ${ϒ}_{e}(t+1)=\Delta \tilde{R}(t+1)$.

The *H*_2_ performance index in Equation (15) can be reformulated as:

(16) $$\begin{array}{cc} J & =E\left\{tr(\Delta \tilde{R}(t+1)\tilde{R}{\left(t+1\right)}^{T}\Delta^{T})\right\}\\ & =tr(\Delta E\left\{\tilde{R}(t+1)\tilde{R}{\left(t+1\right)}^{T}\right\}\Delta^{T}) \end{array}$$

From Equation (16), the covariance matrix of $\tilde{R}(t+1)$ at the steady state is obtained by:

(17) $$\begin{array}{ll} \tilde{R}(t+1) & =R(t+1)-\hat{R}(t+1)\\ & =A_{s}R(t)+B_{s}w_{s}(t)-\left\{A_{s}\hat{R}(t)+L_{2}\left[y(t)-C_{s}\hat{R}(t)\right]\right\}\\ & =A_{s}\tilde{R}(t)+B_{s}w_{s}(t)-L_{2}\left[C_{s}R(t)+D_{s}v_{s}(t)-C_{s}\hat{R}(t)\right]\\ & =A_{s}\tilde{R}(t)+B_{s}w_{s}(t)-L_{2}\left[C_{s}\tilde{R}(t)+D_{s}v_{s}(t)\right]\\ & =(A_{s}-L_{2}C_{s})\tilde{R}(t)+B_{s}w_{s}(t)-L_{2}D_{s}v_{s}(t) \end{array}$$

From Equations (15) and (17), the mean-square error or *H*_2_ performance index can be described as follows:

(18) $$\begin{array}{ll} E\left\{\tilde{R}(t+1)\tilde{R}{\left(t+1\right)}^{T}\right\} & =E\{\left[\left(A_{s}-L_{2}C_{s})\tilde{R}(t)+B_{s}w_{s}(t)-L_{2}D_{s}v_{s}(t\right)\right]\cdot \left[\tilde{R}{\left(t\right)}^{T}{\left(A_{s}-L_{2}C_{s}\right)}^{T}+w_{s}{\left(t\right)}^{T}B_{s}{}^{T}-v_{s}{\left(t\right)}^{T}D_{s}{}^{T}L_{2}{}^{T}\right]\}\\ & =E\{\left(A_{s}-L_{2}C_{s})\tilde{R}(t\right)\tilde{R}{\left(t\right)}^{T}{\left(A_{s}-L_{2}C_{s}\right)}^{T}+B_{s}w_{s}(t)w_{s}{\left(t\right)}^{T}B_{s}{}^{T}+L_{2}D_{s}v_{s}(t)v_{s}{\left(t\right)}^{T}D_{s}{}^{T}L_{2}{}^{T}\}\\ & =(A_{s}-L_{2}C_{s})E\left\{\tilde{R}(t)\tilde{R}{\left(t\right)}^{T}\right\}{\left(A_{s}-L_{2}C_{s}\right)}^{T}+B_{s}B_{s}{}^{T}+L_{2}D_{s}D_{s}{}^{T}L_{2}{}^{T} \end{array}$$

where *R*(*t*), *v_s_*(*t*), and *w_s_*(*t*) are mutually orthogonal, and the covariance matrices of *v_s_*(*t*), and *w_s_*(*t*) are assumed to be the identity matrix as $E\left\{v_{s}(t)v_{s}{\left(t\right)}^{T}\right\}=I_{n\times n}$ and $E\left\{w_{s}(t)w_{s}{\left(t\right)}^{T}\right\}=I_{n\times n}$.

In practical design, the covariance matrix $E\left\{\tilde{R}(t)\tilde{R}{\left(t\right)}^{T}\right\}$ at steady state t→∞ is constant and can be denoted as $E\left\{\tilde{R}(t)\tilde{R}{\left(t\right)}^{T}\right\}=\Psi$. By combining Equations (16) and (18), we have

(19) $$\begin{array}{ll} J & =tr(\Delta E\left\{\tilde{R}(t+1)\tilde{R}{\left(t+1\right)}^{T}\right\}\Delta^{T})\\ & =tr(\Delta \left[(A_{s}-L_{2}C_{s})\Psi {\left(A_{s}-L_{2}C_{s}\right)}^{T}+\Gamma \Gamma^{T}+L_{2}\Theta \Theta^{T}L_{2}{}^{T}\right]\Delta^{T})\\ & =tr(\Delta \left[(A_{s}-L_{2}C_{s})\Psi {\left(A_{s}-L_{2}C_{s}\right)}^{T}-\Psi +B_{s}B_{s}{}^{T}+L_{2}D_{s}D_{s}{}^{T}L_{2}{}^{T}\right]\Delta^{T})\\ & +tr(\Delta \Psi \Delta^{T}) \end{array}$$

It is obvious that mean-square error *J* has an upper bound as below:

(20) $$J\leq tr(\Delta \Psi \Delta^{T})$$

If the inequality as below holds:

(21) $$(A_{s}-L_{2}C_{s})\Psi {\left(A_{s}-L_{2}C_{s}\right)}^{T}-\Psi +B_{s}B_{s}{}^{T}+L_{2}D_{s}D_{s}{}^{T}L_{2}{}^{T}<0$$

Let *P*_2_ = Ψ^−1^ and *Y*_2_ = *P*_2_*L*_2_, and multiply the left and right sides of Equation (21) by a positive definite matrix *P*_2_, then Equation (21) can be expressed as:

(22) $$(P_{2}A_{s}-Y_{2}C_{s})P_{2}^{-1}{\left(P_{2}A_{s}-Y_{2}C_{s}\right)}^{T}-P_{2}+P_{2}B_{s}B_{s}{}^{T}P_{2}+Y_{2}D_{s}D_{s}{}^{T}Y_{2}^{T}<0$$

For obtaining the solution *P*_2_ of Equation (22), the famous Schur complement is applied to Equation (22) to transfer the inequality of Equation (22) into an equivalent linear matrix inequality (LMI) [18] form as below:

(23) $$\left[\begin{array}{cccc} P_{2} & P_{2}B_{s} & Y_{2}D_{s} & \left(P_{2}A_{s}-Y_{2}C_{s}\right)\\ B_{s}{}^{T}P_{2} & I & 0 & 0\\ D_{s}{}^{T}Y_{2}^{T} & 0 & I & 0\\ {\left(P_{2}A_{s}-Y_{2}C_{s}\right)}^{T} & 0 & 0 & P_{2} \end{array}\right]>0$$

Then, *P*_2_ and *Y*_2_ of Equation (23) can be numerically calculated by using the LMI toolbox of Matlab software [19] simultaneously, and the optimal estimation gain *L*_2_ = *P*_2_^−1^ *Y*_2_.

From the above mathematical derivations for the speech enhancement problem of mobile devices with a dual microphone array, the overall design procedure of the proposed *H*_2_ estimator can be summarized as follows:

Step 1. Assume the covariance matrices of *v_s_*(*t*) and *w_s_*(*t*) are identity matrices, and ∆ is a constant matrix.
Step 2. Solve the LMI in Equation (23) for getting the matrices *P*_2_ and *Y*_2_.
Step 3. Calculate the estimation gain *L*_2_ = *P*_2_^−1^ *Y*_2_.
Step 4. Construct the optimal *H*_2_ estimator by Equation (13) as below
  $$\begin{array}{l} \hat{R}(t+1)=A_{s}\hat{R}(t)+L_{2}\left[y(t)-C_{s}\hat{R}(t)\right]\\ \;\hat{ϒ}(t)=\Delta \hat{R}(t) \end{array}$$

## 3. Practical Implementation and Performance Verification

As is mentioned above, a speech enhancement design for mobile devices with a dual microphone array is investigated. In this section, the practical performance of this proposed method will be assessed for two testing scenarios and three performance indices: (1) final cross-correlation between the estimated and real clean speech, (2) enhanced signal-to-noise ratio (*E-SNR*), and (3) perceptual evaluation of speech quality (PESQ). Before the verification of this proposed speech enhancement design, brief descriptions of *E-SNR*, PESQ, the installation of the practical experiment environment and related instruments will be introduced.

### 3.1. Enhanced SNR and Perceptual Evaluation of Speech Quality

(i) The *E-SNR* with an estimation design can be expressed as:

*E-SNR = Denoised SNR − Original SNR* (24)

where *Denoised SNR* is the SNR of the treated speech signal and *Original SNR* is the SNR of speech signal without any treatment.

(ii) PESQ which combines the advantages of perceptual analysis/measurement system (PAMS) and perceptual speech quality measure (PSQM) is a test methodology for automated objective assessment of speech quality to replace traditional jury test. The range of PESQ is 1 to 4.5, where 1 is the worst quality and 4.5 is the best quality.

### 3.2. Practical Implementation and Performance Verification

**Figure 3:**
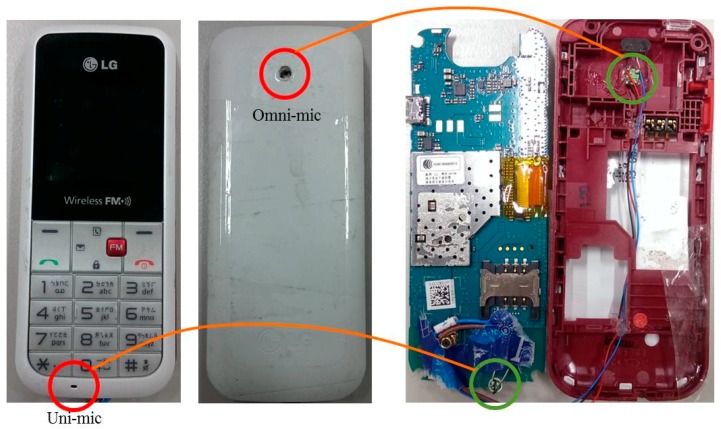
Illustration of the dual microphone array in the mobile device.

From the inner and outer illustration of the mobile device in Figure 3, the omnidirectional microphone (Omni-Mic) is installed in the back of the mobile device, and the unidirectional microphone (Uni-Mic) is placed in the front side and is close to the mobile device user’s mouth.

**Figure 4:**
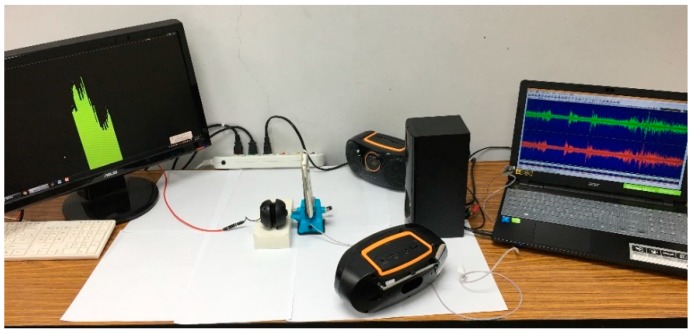
Installation of the test environment of the proposed speech enhancement design.

The overall assessment environment of the speech enhancement performance is set up as Figure 4. In this installation, a 20 s long phrase (clean speech) is broadcasted from a laptop in front of the mobile device. Without loss of generality, a testing environment with multiple directional background noises is used to verify the speech enhancement performance of this proposed method. In this testing arrangement, three trumpets loudly broadcast background noises simultaneously from different directions (0°, 45°, −45°). The relative distance is 4 cm far from the mobile device to the clean speech, and the relative distance from each of background noises to the mobile device is 20 cm. This testing arrangement is a standard and customized specification in the function test of all delivered speech enhancement designs of mobile devices.

### 3.3. Initialization of the Practical Realization

The system order *n* of the whitened speech model in Equation (4) is chosen as 10 for saving calculation power in the following speech enhancement assessments. Key parameters of the proposed estimator are given as Table 1 and Table 2.

Two various scenarios will be utilized to verify the speech enhancement ability of this proposed design. The user’s testing speech is an English phrase, and for the purpose of assessing the robustness of this proposed design, two English songs: One sang by a female singer and one sang by a male singer, are used as background noises.

### 3.4. Practical Test of This Proposed Method for a Phrase

**Scenario 1:** A mobile device user who says “Smart phones become more and more popular in recent years, and the performance of smart phones has gotten better and better. Unfortunately, in a noisy environment, the communication quality is still awful and no suitable solution exists for it” is used as the clean speech in communication, and the English song: “Let it go” sang by a female singer Idina Menzel is used as the background noise.

**Figure 5:**
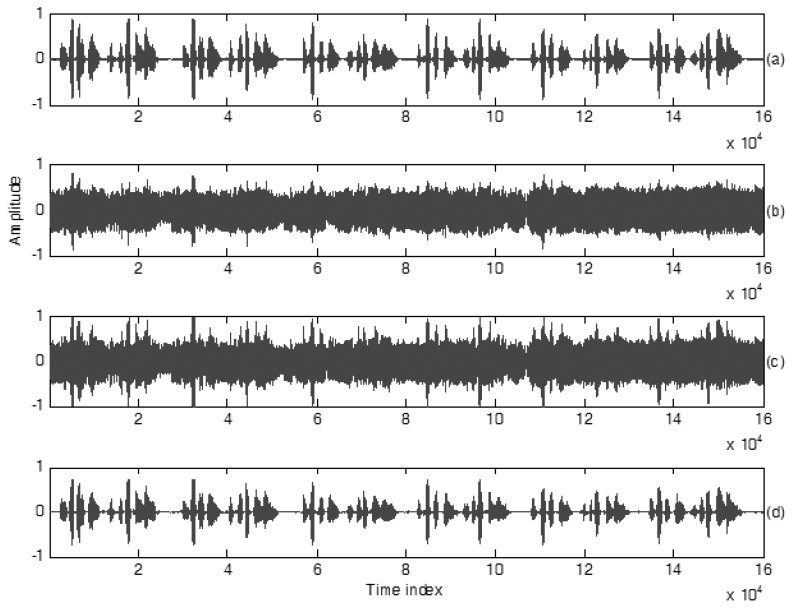
Waveform plots of speech signals, (**a**) clean speech, PESQ = 4.5, spoken by a speaker in Scenario 1, (**b**) recorded speech using Omni-microphone, PESQ = 1.34, (**c**) recorded speech using Uni-microphone, PESQ = 1.42, (**d**) enhanced speech using the proposed method, PESQ = 3.78.

Without any speech enhancement treatments, the original SNR for this testing scenario is −5.2 dB. Clean speech, two sound signals measured by Uni and Omni Mics and the estimated result are revealed in Figure 5. Two original signals measured by Uni and Omni Mics are plotted in subfigures (b) and (c) of Figure 5. The estimation result of the proposed *H*_2_ estimator design is shown in the subfigure (d) of Figure 5. From subfigures (b) and (c), the output signal of Uni-Mic shows us a bigger amplitude than that of Omni-Mic, and the output signal shapes of Uni-Mic and Omni-Mic are similar. The clean speech is too weak to be recognized by only using Uni-Mic in practice, even the user’s mouth is close to Uni-Mic due to the background noise being too loud. Comparisons are made between the signal measured by Uni-Mic and the proposed method, and the contrast of the estimated speech in Figure 5d with respect to the original clean speech in Figure 5a. Obviously, the proposed method provides promising speech enhancement performance when the mobile device user is subject to very noisy circumstances. The final assessments are: cross-correlation 0.91, *E-SNR* 18.3 dB and PESQ = 3.78.

**Figure 6:**
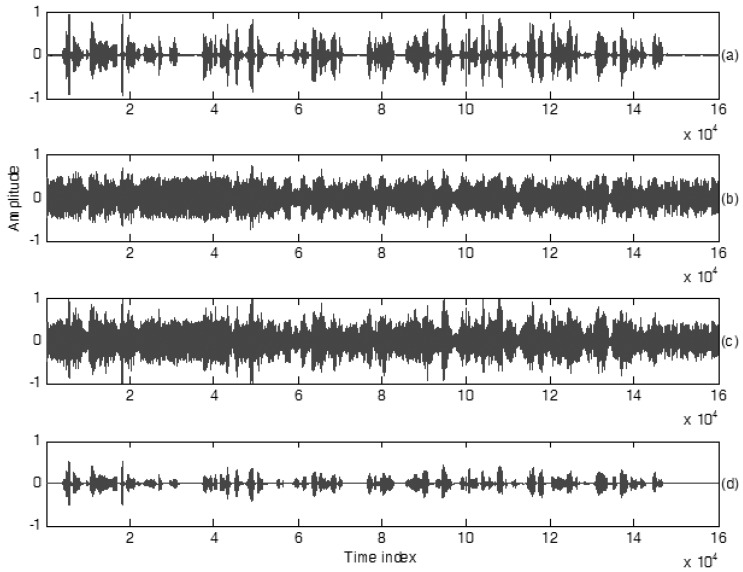
Waveform plots of speech signals, (**a**) clean speech, PESQ = 4.5, spoken by a speaker in Scenario 2, (**b**) recorded speech using Omni-microphone, PESQ = 1.52, (**c**) recorded speech using Uni-microphone, PESQ = 1.61, (**d**) enhanced speech using the proposed method, PESQ = 3.84.

**Scenario 2:** In this scenario, the clean speech is the same as **Scenario 1**, however the background noise is an English song: “Free loop” sang by the male singer Daniel Powter. The original SNR for this testing scenario is −5.3 dB. The clean speech is completely covered by this English song. For published speech enhancement designs, recovering the original speech from such an un-ideal situation is actually a challenging task. The subfigures (b) and (c) of Figure 6 are the measured signals of Uni Mic and Omni Mic, respectively. The practical estimation result of the clean speech is shown as the first subfigure of Figure 6. From the comparisons of the estimated speech and the measured signal of Uni Mic plotted in Figure 6c,d, most background noises are obviously removed before speech transmission of the mobile device. From the estimation result revealed in Figure 6d, it is quite similar to the original clean speech in Figure 6a. In this scenario, the cross-correlation is 0.92, *E-SNR* is 18.6 dB, and PESQ is 3.84. Table 3 lists the *E-SNRs*, the final cross correlations of the estimated results of this proposed method to with respect to clean speeches and PESQs. From Table 3, the average *E-SNRs* reaches 18.45 dB, the similarity calculated by the cross-correlation index is 0.915 and average PESQ is 3.81. These three indexes are superior to the existing speech enhancement designs which are with the average 10 dB *E-SNR*. Speech enhancement performances of these two scenarios show us that our proposed method possesses robustness property corresponding to various background noises (different sex).

**Figure 7:**
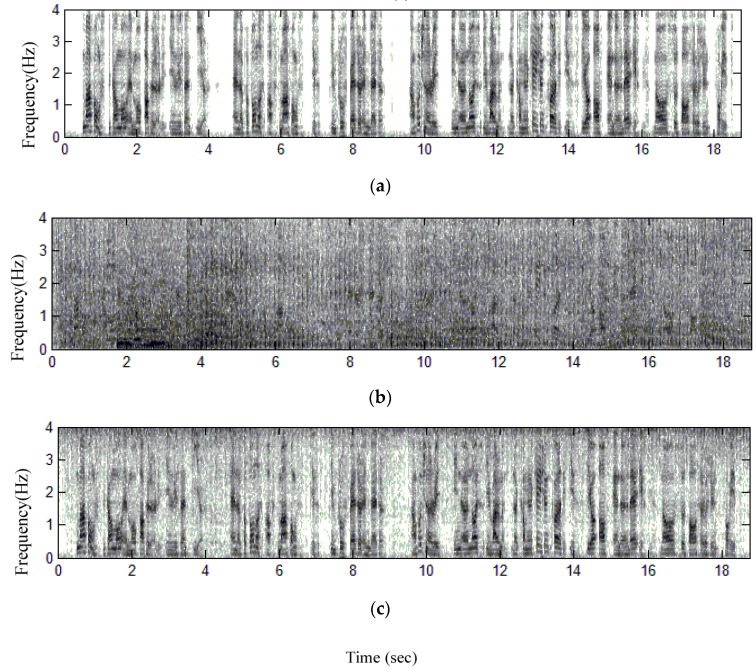
Spectrograms of speech signals for Scenario 1, (**a**) clean speech spoken by a speaker in Scenario 1, (**b**) recorded speech using Omni-Mic, (**c**) enhanced speech using the proposed method.

**Figure 8:**
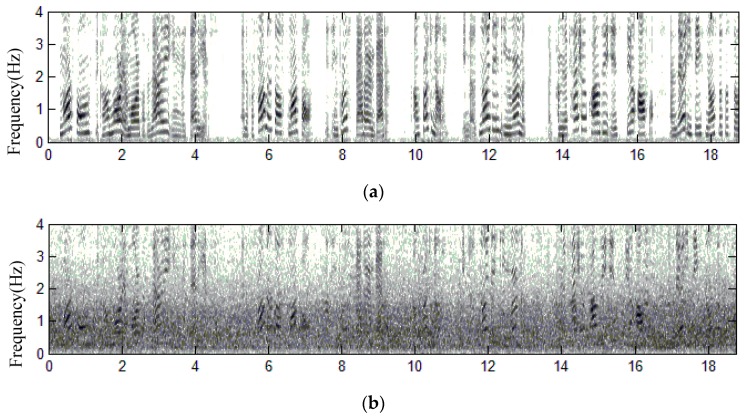

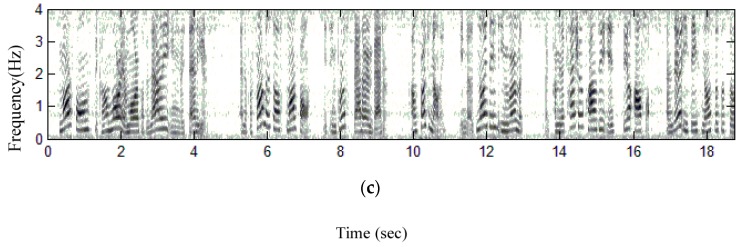
Spectrograms of speech signals for Scenario 2, (**a**) clean speech spoken by a speaker in Scenario 2, (**b**) recorded speech using Omni-Mic, (**c**) enhanced speech using the proposed method.

**Spectrograms**: In order to yield more information about the residual noise and speech distortion, we analyze the time-frequency distribution of the enhanced speech and evaluate the structure of residual noise by observing speech spectrograms. Figure 7 and Figure 8 present comparisons of the spectrograms for enhanced speech in Scenarios 1 and 2, respectively. Clean speech signals are heavily corrupted by non-stationary background noises with very low-SNR environments, where SNR = −5.2 dB in Figure 7 and −5.3 dB in Figure 8, respectively. Speech signals heavily corrupted by various kinds of non-stationary background noises is shown in Figure 7b and Figure 8b. Observing the spectrograms of enhanced speech shown in Figure 7c and Figure 8c, the harmonic spectra of vowel signals can be well preserved in the enhanced speech signals. As a result, the proposed method does not suffer from the over-attenuation on noisy speech when removing a quantity of background noise. In addition, the spectrograms also reveal the fine structure of spectra in speech-activity regions. A muffled signal is absent in the enhanced speech. The proposed method is superior in the removal of background noise during speech-pause regions. Only a little quantity of residual noise exits, so the enhanced speech sounds are not annoying to the human ear. This proves that the performance of the proposed method is satisfied.

## 4. Conclusions

In this investigation, an integrated speech enhancement design which can effectively cancel background noises surrounding the used mobile devices is proposed. Firstly, a dual microphone array is installed in mobile devices for collecting a pair of speech patterns for the objective of whitening the speech, which is corrupted by colored background noises. The slightly noised speech is then mathematically modelled as a difference equation which is driven by a white noise and expressed as a vector-matrix formulation. For mitigating residual unknown and random background noises, an *H*_2_ estimator is derived based on the vector-matrix formulation. By applying this well-developed design to a real mobile device platform, the average enhanced SNR is more than 18 dB, and an average value 0.915 in the similarity can be obtained for the original clean speeches and estimated results. From the results with respect to various testing scenarios, this proposed method actually delivers a satisfactory speech enhancement performance for the speech communication applications of mobile devices.

## Figures and Tables

**Table 1:** Parameters of the proposed *H*_2_ estimator.

Parameter	Description	Value
$\hat{R}(t)$	Initial values of estimator states	$0_{n\times 1}$
P	The coefficient covariance	$I_{n\times n}$
Δ	Constant matrix	$I_{n\times n}$

**Table 2:** Estimation gain *L*_2_ and *P*_2_ of *H*_2_ estimator in steady state condition.

$L_{2}=\left[\begin{array}{l} 0.9751\\ 0.9778\\ -6.4285e^{-5}\\ -3.5771e^{-6}\\ -2.5308e^{-6}\\ 3.5226e^{-6}\\ -3.5417e^{-6}\\ 1.9277e^{-6}\\ 2.5554e^{-6}\\ -2.8470e^{-6} \end{array}\right]$, $P_{2}={\left[\begin{array}{ccccc} 0.0202 & 0 & \cdots & \cdots & 0\\ 0 & 0.0202 & \ddots & \ddots & \vdots \\ \vdots & \ddots & \ddots & \ddots & \vdots \\ \vdots & \ddots & \ddots & 0.0202 & 0\\ 0 & \cdots & \cdots & 0 & 0.0202 \end{array}\right]}_{10\times 10}$

**Table 3:** *E-SNR*s and the final cross-correlations for **Scenario 1** and **Scenario 2**.

	Type	Final Cross-Correlation	*E-SNR*	PESQ
**Scenario 1**	Pure Speech: A Phrase	0.91	18.3 dB	3.78
	English Song: Let it go, female singer Idina Menzel			
**Scenario 2**	Pure Speech: A Phrase	0.92	18.6 dB	3.84
	English Song: Free loop, male singer Daniel Powter			
